# Effects of* Crinum jagus* Water/Ethanol Extract on* Shigella flexneri*-Induced Diarrhea in Rats

**DOI:** 10.1155/2019/9537603

**Published:** 2019-03-12

**Authors:** Paul Aimé Noubissi, Michel Archange Fokam Tagne, Gaëtan Olivier Fankem, Joseph Ngakou Mukam, Henri Wambe, René Kamgang

**Affiliations:** ^1^Department of Zoology and Animal Physiology, Faculty of Science, University of Buea, Cameroon; ^2^Department of Biological Science, Faculty of Science, University of Ngaoundéré, Cameroon; ^3^Animal Physiology Laboratory, Faculty of Science, University of Yaoundé I, Cameroon; ^4^Department of Biological Science, Faculty of Science, University of Dschang, Cameroon; ^5^Laboratory of Endocrinology and Radioisotopes, Institute of Medical Research and Medicinal Plants Studies (IMPM), Yaoundé, Cameroon

## Abstract

Diarrheal disease, characterized by the release of more than three loose or liquid stools per day, remains one of the leading causes of morbidity and mortality in children below 5 years of age in developing countries. Many drugs used in diarrhea management face contraindication and, with regard to infectious diarrhea, resistance of some bacterial strains; this therefore increases the need of new alternative and more effective drugs. This study aimed to evaluate anti-*Shigella flexneri* activities of* Crinum jagus* water/ethanol extract.* In vitro* activities were assayed by disc diffusion and agar dilution methods and i*n vivo* section on* Shigella flexneri*-induced diarrhea in rats. This was done by oral administration of 9 X 10^8^ CFU of* Shigella flexneri* to rats that were treated twice daily with* Crinum jagus* water/ethanol extract for seven consecutive days. Ciprofloxacin was used as positive control. Daily* Shigella flexneri* load was evaluated. After one treatment week, animals were then sacrificed and interleukins (IL-2 and INF-*γ*), immunoglobulins (IgA and IgM), motilin, vasoactive intestinal peptide, and ions (sodium, potassium, calcium, and chloride) levels were determined. Also, blood cell count was realized.* Crinum jagus* water/ethanol extract dose-dependently inhibited* Shigella flexneri* growth with inhibition diameter of 18.90 and 25.36 mm, respectively, at 0.39 and 200 mg/mL. Minimum inhibitory concentration (MIC) was 0.10 mg/mL and minimum bactericidal concentration (MBC) was 0.30 mg/mL with MBC/MIC ratio of 3.0. In* Shigella flexneri*-induced diarrheic rats,* Crinum jagus* reduced (p<0.01) diarrheal stools emission and* Shigella* load and lowered IL-2, INF-*γ*, IgA, IgM, and motilin blood levels, whereas it increased (p<0.01) vasoactive intestinal peptide, sodium, potassium, calcium, and chloride blood levels. In diarrheal rats,* Crinum jagus* restored the decreasing white blood cells and haemoglobin and restored the damaged colon epithelium, where it reduced the density of mucus-filled goblet cells. These results confirm the use of* Crinum jagus* in ethnomedicine in diarrhea treatment.

## 1. Introduction

Diarrhea is the emission of more than three soft or liquid stools in 24 h [1]. It can last few days to few weeks and is at the origin of the body dehydration and the loss of mineral salts necessary for survival. Diarrhea can be provoked by an increased motility of the digestive tract, increased secretion, and/or decreased fluid absorption, resulting in electrolytes (Na^+^, K^+^, and Cl^−^) and water loss [1]. Diarrheal diseases, which are the second leading cause of mortality in children below five years of age, are responsible for more than 3 million deaths each year worldwide [2]. According to World Health Organization (WHO), in developing countries, each infant yearly experiences about three episodes of diarrhea; and about 1.5 billion cases of diarrhea occur each year in developing countries, resulting in 5.483 deaths every day [3]. In Cameroon, diarrhea is the main cause of infant mortality and ranks third in morbidity with prevalence of 13.6% across the country [3]. Despite the declining diarrheal mortality rates, diarrheal diseases are still considered as one of the principal causes of childhood morbidity and mortality in developing countries [4]. They kill more young children around the world than tuberculosis, acquired immune deficiency syndrome (AIDS), and malaria combined [5]. Diarrhea is sometimes caused by microbial agents (bacteria, viruses, and/or parasites) colonizing the digestive tract. Other etiologies include food poisoning, antibiotics, laxative agents, or certain medical conditions. With other infectious diseases, diarrhea will remain a source of concern for global health over the next 20-30 years.

Among infectious diarrhea, shigellosis continues to wreak havoc around the world, with a high infectivity rate. It is the main cause of bloody diarrhea worldwide and is endemic in most developing countries, causing more than 164.7 million episodes of bloody diarrhea and about 700 000 deaths per year [6]. Ninety nine percent (99%) of shigellosis occurs in developing countries with most cases (about 70%) and deaths (about 60%) in children below five years of age [7]. In human, 10 to 200* Shigella* organisms are enough to induce shigellosis [8]. In most diarrhea cases, especially in malnourished infants or in immune deficiency, patients die actually from severe dehydration with electrolyte imbalance [9]. Oral rehydration therapy has been at the center of the effective diarrheal diseases management. It often fails, however, in high stool output condition. Furthermore, although currently used drugs are important in diarrhea management, they are still associated with adverse effects and contraindications [10]. Different antibiotics are effective in the management of infectious diarrhea but they usually face resistance developed by some microbial strains [11, 12]. In addition, diarrheal diseases management cost is high, as is the unavailability or toxicity of conventional drugs, which increases the need for alternative medicines based on medicinal plants available locally for the treatment of these diseases [13].

*Crinum jagus* (Amaryllidaceae) is a plant widely used in African folk medicine as antitumor, antiviral, antiparasitic, and immunostimulator plant and against mental trouble and snake bite [14]. According to traditional healers in the Western Region of Cameroon (Central Africa),* Crinum jagus *is used against poison and in the treatment of gastrointestinal diseases [15]. In previous studies,* Crinum jagus* ethanol/water extract administered in rats at 25, 50, and 100 mg/kg BW showed significant antidiarrheal and antisecretory activity on castor oil-induced diarrhea [9]. Phytochemical study of this extract showed the presence of volatile oils, fatty acids, catechic tannins, carbohydrates, alkaloids, sterols, triterpenes, flavonoids, coumarins, anthocyanidins, polyphenols, and saponins [15]. This study thus aimed to investigate the antidiarrheal potential of ethanol/water crude extract of* Crinum jagus* on* Shigella flexneri*-induced diarrhea in albino rats.

## 2. Materials and Methods

### 2.1. Plant Material

Fresh* Crinum jagus* plants were collected from Batié (West Region, Cameroon, harvesting coordinates 9°25′17′′N and 13°27′2′′E) in August 2012. The study site did not involve endangered or protected species. The plant specimen was identified at the Yaounde National Herbarium, in comparison with the specimen referenced HNC 14049. The whole plant was cleaned, reduced into small pieces, dried in the shade, and then crushed to get a powder. The plant powder was extracted with ethanol/water mixture (1:4). For this, 1 kg of powder was macerated for 48 h in 5 L (1 L of ethanol/4 L of water) at room temperature and then filtered with N° 1 Whatman filter paper. The residue obtained was further macerated for 48 h in the same solvent (5 L) and then filtered. Likewise, the 2 filtrates obtained were pooled and concentrated in a rotary evaporator (BÜCHI Rota vapor R-124) at 45°C to obtain 291.6 g of dry dark hydroethanolic extract which was stored at +4°C in a well-closed container until use. Extract solutions were prepared with distilled water before oral administration to rats or for* in vitro* tests.

### 2.2. Experimental Animals

Healthy* Wistar* albino rats (110–150 g) of either sex obtained from the animal house of the University of Buea, Faculty of Science, Department of Zoology and Animal Physiology, were used. Animals housed in clean metabolic cages (1 rat/cage) had free access to water and standard rat diet consisting of carbohydrates 50-55%, fats 15-20%, and proteins 25-30% [16].* In vivo* experiments were performed following the European Union Guidelines on Animal Care (Council EEC 86/609) [17] adopted in Cameroon by the Institutional Committee of the Ministry of Scientific Research and Innovation.

### 2.3. Microbial Strains

The microbial strain,* Shigella flexneri*, used in this study was obtained from the Centre Pasteur of Yaoundé, Cameroon, and was isolated from local patients.

### 2.4. In Vitro Antimicrobial Studies: Antimicrobial Susceptibility

The susceptibility screening of* Crinum jagus* ethanol/water extract was performed by the disk diffusion method [18]. Muller-Hinton agar medium (Diagnostic Liofilchem, Italy, Ref 610033) was autoclaved (121°C, 15 min) and poured into sterile petri dishes (4 mL/petri dish, 3-4 mm depth). After solidification, culture media were inoculated with 1 mL of* Shigella flexneri* suspensions at 5.0 X 10^5^ CFU/mL. Filter paper discs of 6 mm diameter were impregnated with 10 *μ*L of plant extract at various dilutions (0.39, 0.78, 1.56, 3.12, 6.25, 12.50, 25.00, 50.00, 100.00, and 200.00 mg/mL) or with ciprofloxacin (RYAN PHARMA UK; 30 *μ*g/mL) and placed on the surface of the inoculated Muller-Hinton agar. The inoculated plates were kept at room temperature for 1 h and then incubated at 37°C during 24 h for antibacterial activity. Each assay in this experiment was replicated three times [19].

### 2.5. *In Vitro* Antimicrobial Studies: Minimal Inhibitory Concentration (MIC)

Ciprofloxacin (1.0 X 10^−3^ to 15.0 X 10^-3 ^mg/mL) was used as standard for antibacterial activities. Minimum inhibitory concentration is the lowest concentration with no visible microbial growth [20]; it was determined by agar dilution technique [21, 22]. All inhibition assays were conducted in triplicate. A solution set of extract of concentrations ranging from 50.00 to 0.05 mg/mL was prepared by dilution and 2 mL aliquot of each extract solution was added to 18 mL of presterilized molten Muller-Hinton agar or to 18 mL of Salmonella Shigella Agar (SS Agar, Titan Biotech Ltd. India 386) at 40°C giving a final concentration ranging from 5.00 to 0.005 mg/mL.

The medium in tubes was then allowed to solidify. The surfaces of the media were streaked with 18-hour-old* Shigella flexneri* cultures, and the tubes were later incubated for 24 h at 37°C. After 24 h of incubation, the tubes were observed and the first in the series (ascending extract or antibiotic concentrations) not showing visible growth was considered as the MIC [23].

### 2.6. *In Vitro* Antimicrobial Studies: Minimum Bactericidal Concentration (MBC) Assessment

To determine the minimum bactericidal concentration of the plant extract against* Shigella flexneri*, the tubes that showed no microbial growth were subcultured by streaking with a platinum loop on sterile Muller Hinton agar plates and incubated for 24 h at 37°C. The MBC values were considered as the lowest concentrations of the extract not showing any microbial growth on the agar plates [18]. All antimicrobial tests were performed under strict aseptic conditions.

### 2.7. *In Vivo* Antimicrobial Studies: Shigella flexneri-Induced Diarrhea in Rats

Before experimentation, all rats were deparasitized by daily administration (6:00 AM and 6:00 PM) of tetracycline (tetracycline HCl, New Divine Favour Pharma IND. LTD., Nigeria) 10 mg/kg BW for 3 consecutive days. Four days after this deparasitization, animals were placed separately in individual cages and received* per os*, except the normal control group, an oral administration of 9.0 X 10^8^ CFU/mL of* Shigella flexneri* corresponding to a 3.0 McFarland turbidity standard [19].

Soon after the appearance of the first diarrheal stools (approximately 24 h after* Shigella flexneri* administration), diarrheic rats were divided into five groups of five rats each:

(i) Diarrheic control group (DC) receiving distilled water (10 mL/kg BW)

(ii) Positive control group (Cipro) receiving ciprofloxacin (RYAN PHARMA, UK) 2.5 mg/kg BW

(iii) Three test groups treated with* C. jagus* water/ethanol extract of 25 (WECj25), 50 (WECj50), or 100 (WECj100) mg/kg BW.

Animals were treated twice daily for 7 days and, each day, their behavior and weight variation were evaluated as well as* Shigella flexneri* density in the stools. For this, 0.5 g of fresh stools collected from each diarrheic rat was dissolved in 4.5 mL sterile physiologic solution. After homogenization, 250 *μ*L of the obtained stools solution was further diluted in 9.750 mL of sterile physiologic solution. 50 *μ*L of this final solution was finally taken and cultured on SS agar for 24 h at 37°C.* Shigella* density was then determined by direct count of colonies number [24]. After treatment period, rats were sacrificed and blood was collected for spectrophotometric determination of ions (sodium, potassium, calcium, and chloride) levels, for the determination of interleukin-2 (IL-2), interferon gamma (INF-*γ*), immunoglobulins (IgA and IgM), motilin, and vasoactive intestinal peptide levels using ELISA method as well as for blood cell count using hematology analyzer (Golden Harvest Industries BC 2800 Hematology Analyzer). Colon was used for histological studies.

### 2.8. Acute Toxicity

The acute oral toxicity of* C. jagus* water/ethanol extract was carried out using the limit test recommendations of OECD 425 Guideline. One healthy adult female Wistar rat was fasted (with free access to water) for 18 h and then orally loaded with 5000 mg/kg of the extract. The rat was then strictly observed during 14 days for general signs and symptoms of toxicity (locomotion, aggressiveness, nature of the fur, quality of the stools, marked pain, and distress). After this observation period, since the animal survived, two additional female rats received the same treatment [25].

## 3. Results

### 3.1. Antimicrobial Susceptibility

*Crinum jagus *water/ethanol extract inhibited* Shigella flexneri *growth (Table 1). The antimicrobial activity was dose-dependent with an inhibition diameter of 25.36 and 18.90 mm, respectively, at 200 and 0.39 mg/mL. Ciprofloxacin (30 *μ*g/mL) was very effective against* Shigella flexneri* with an inhibition diameter of 28.0 mm.

### 3.2. Minimal Inhibitory Concentration and Minimum Bactericidal Concentration (MBC)

The water/ethanol extract and ciprofloxacin showed* in vitro* inhibitory activity on* Shigella flexneri *growth. The minimum inhibitory concentrations (MICs) were, respectively, 0.10 and 2.0 X 10^-3 ^mg/mL; and the minimum bactericidal concentrations were 0.30 and 4.0 X 10^-3 ^mg/mL, respectively (Table 2). MBC/MIC ratios were 3.0 and 2.0, respectively, for* Crinum jagus* and ciprofloxacin.

### 3.3. Effect of* Crinum jagus* Water/Ethanol Extract on Rats'* Shigella flexneri* Load

**Figure 1:**
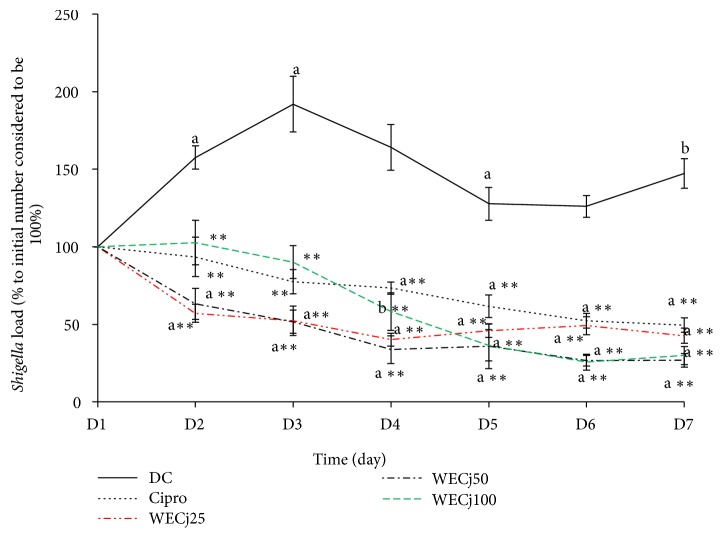
*Shigella flexneri* load in rat stools after 7 days of treatment with* C. jagus* water/ethanol extract of 25 (WECj25), 50 (WECj50), and 100 mg/kg (WECj100) and ciprofloxacin 2.5 mg/kg (Cipro) (n =5). Significant difference: *∗∗*p<0.01 compared to diarrheic control (DC); ^a^p<0.01 and ^b^p<0.05 compared to the initial bacterial load.

In diarrheic control rat stools (DC),* S*.* flexneri* load increased from the first day of onset of diarrhea stools: +57 and +92% (p<0.01), respectively, on the second and third day as compared to the initial bacterial load. In treated groups, bacterial load significantly (p<0.01) decreased (Figure 1). The number of* S*.* flexneri* in rats treated with* C. jagus* water/ethanol extract significantly decreased (p<0.01) from the first to the seventh day of treatment. On the seventh day, bacterial load as compared to the initial load was 42, 26, 29, and 50%, respectively, for* C. jagus* water/ethanol extract at 25, 50, or 100 mg/kg and for ciprofloxacin 2.5 mg/kg (Figure 1).

### 3.4. Effect of* Crinum jagus* Water/Ethanol Extract on* Shigella flexneri* Diarrheic Rats' Body Weight Variation

**Figure 2:**
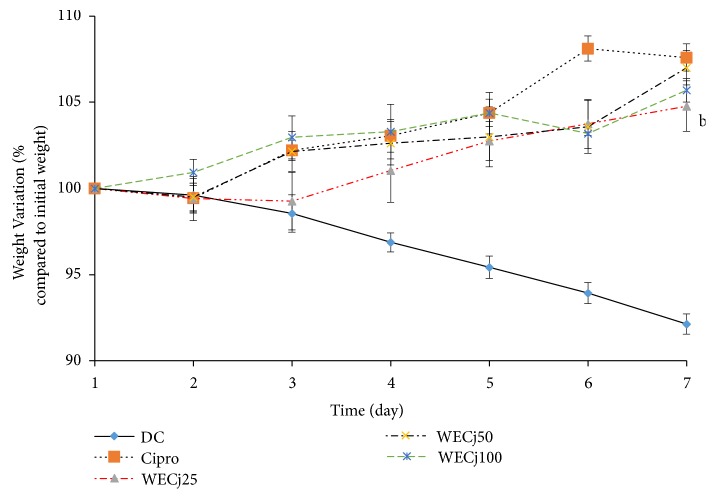
Mean body weight variation in* Shigella flexneri*-induced diarrhea in rats during 7 days' administration of water,* C. jagus *water/ethanol extract of 25 mg/kg (WECj25), 50 mg/kg (WECj50), and 100 mg/kg (WECj100), and ciprofloxacin 2.5 mg/kg (Cipro) (n =5). Significant difference: ^b^p<0.05 compared to the initial weight.

After the onset of diarrhea, we observed a weight decrease in diarrheic control animals. After seven treatment days, weight loss was about 8.0% compared to the initial weight. On the other hand, treated animals presented slight weight gain during the treatment period. The increase as compared to the initial weight was 5.0, 7.0, 6.0, and 8.0% at 7th day, respectively, with* C. jagus* water/ethanol extract (WECj25, WECj50, and WECj100) and ciprofloxacin 2.5 mg/kg (Figure 2).

### 3.5. Effect of* Crinum jagus* Water/Ethanol Extract on* Shigella flexneri* Diarrheic Rats' Interleukin-2 and Interferon Gamma Plasma Levels

**Figure 3:**
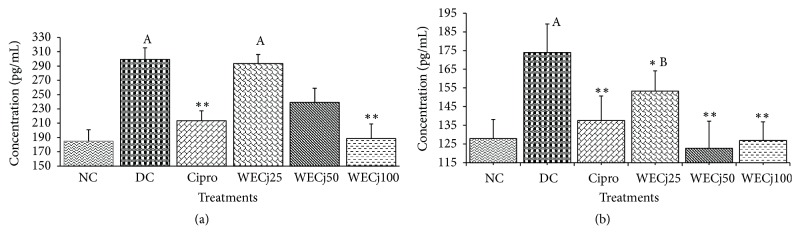
Concentration of interleukin-2 (a) and interferon-*γ* (b) in diarrheic rats treated with* C. jagus* water/ethanol extract of 25 mg/kg (WECj25), 50 mg/kg (EWCj50), and 100 mg/kg (EWCj100) and ciprofloxacin 2.5 mg/kg (Cipro) (n =5). Significant difference: *∗∗*p<0.01 compared to diarrheic control (DC); ^A^p<0.01 and ^B^p<0.05 compared to normal control (NC).

Interleukin-2 level significantly increased (p<0.01) in diarrheic control group compared to normal rats: 299.35 ± 16.18 pg/mL against 184.70 ± 16.22 pg/mL. Interleukin-2 concentrations were 213.56 ± 13.77, 293.63 ± 12.57, 238.95 ± 19.84, and 188.49 ± 20.31 pg/mL, respectively, in group treated with ciprofloxacin and* C. jagus* water/ethanol extract of 25, 50, or 100 mg/kg BW, respectively (Figure 3(a)).* S. flexneri* administration induced a significant (p<0.01) increase of interferon-*γ* plasma level (Figure 3(b)). During one week of* C. jagus* water/ethanol extract treatment, plasma concentration of this cytokine significantly reduced. It was 173.92 ± 15.34 pg/mL in diarrheic control group and 137.51 ± 13.15, 153.32 ± 10.76, 122.73 ± 14.39, and 126.82 ± 10.03 pg/mL in groups treated with ciprofloxacin and* C. jagus* water/ethanol extract of 25, 50, and 100 mg/kg, respectively (Figure 3(b)).

### 3.6. Effect of* Crinum jagus* Water/Ethanol Extract on* Shigella flexneri* Diarrheic Rats' Immunoglobulins A and M Plasma Levels

**Figure 4:**
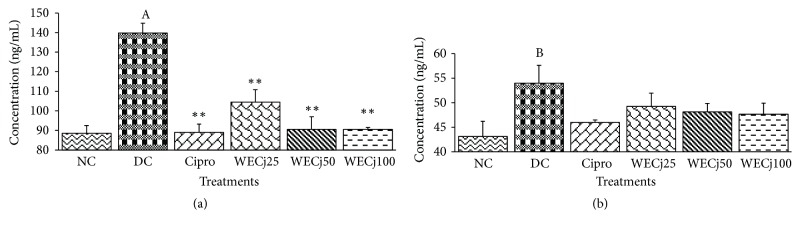
Concentration of immunoglobulins A (a) and M (b) in diarrheic rats treated with* C. jagus* water/ethanol extract of 25 mg/kg (WECj25), 50 mg/kg (EWCj50), and 100 mg/kg (EWCj100) and ciprofloxacin 2.5 mg/kg (Cipro) (n =5). Significant difference: *∗∗*p<0.01 compared to diarrheic control (DC); A: p<0.01 and B: p<0.05 compared to normal control (NC).

Normal rat IgA plasma level was 88.50 ± 3.98 ng/mL.* S. flexneri* administration increased IgA level in diarrheic control group (139.77 ± 5.03 ng/mL). Treatments with 25, 50, or 100 mg/kg of the extract significantly (p<0.01) reduced IgA level: 104.42 ± 6.34, 90.55 ± 6.36, and 90.47 ± 1.14 ng/mL, respectively (Figure 4(a)). In rats treated with ciprofloxacin, IgA plasma level was 88.95 ± 4.28 ng/mL. Diarrheic control rat IgM level significantly increased (p<0.01) compared to normal ones (43.12 ± 3.08 against 53.97 ± 3.60 ng/mL, respectively, for normal and diarrheic control rats). Ciprofloxacin or* C. jagus* water/ethanol extract (25, 50, or 100 mg/kg) treated groups showed reduction in IgM plasma level: 45.97 ± 0.51, 49.23 ± 2.73, 48.10 ± 1.74, and 47.66 ± 2.24 ng/mL, respectively (Figure 4(b)).

### 3.7. Effect of* Crinum jagus* Water/Ethanol Extract on* Shigella flexneri* Diarrheic Rats' Vasoactive Intestinal Peptide (VIP) and Motilin Levels

**Figure 5:**
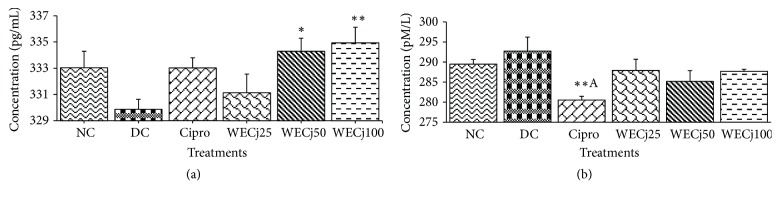
VIP (a) and motilin (b) plasma concentrations in normal rats and in diarrheic rats treated with* C. jagus* water/ethanol extract of 25 mg/kg (WECj25), 50 mg/kg (WECj50), and 100 mg/kg (WECj100) and ciprofloxacin 2.5 mg/kg (Cipro) (n =5). Significant difference: *∗*p<0.05 and *∗∗*p<0.01 compared to diarrheic control (DC); ^A^p<0.01 compared to normal control (NC).

*Shigella flexneri* infection brought a significant (p<0.01) decrease of VIP (Figure 5(a)) but no significant effect on motilin (Figure 5(b)) plasma levels. VIP concentration was 333.03 ± 1.26 and 329.87 ± 0.77 pg/mL, respectively, in normal control and diarrheic control groups against 333.02 ± 0.78, 331.13 ± 1.41, 334.29 ± 1.00, and 334.93 ± 1.19 pg/mL in groups treated with ciprofloxacin or water/ethanol* C. jagus* extract of 25, 50, and 100 mg/kg (Figure 5(a)).

### 3.8. Effect of* Crinum jagus* Water/Ethanol Extract on* Shigella flexneri* Diarrheic Rats' Ionic Parameters

Diarrheic control rats showed an important decrease of Na^+^, K^+^, Ca^++^, and Cl^−^ plasma levels: respectively, 93.33 ± 4.08, 3.75 ± 0.08, 2.44 ± 0.03, and 95.74 ± 1.46 against 143.33 ± 4.08, 5.11 ± 0.09, 3.25 ± 0.01, and 113.83 ± 2.29 mmol/L in normal control.* Crinum jagus* water/ethanol extract (100 mg/kg) significantly (p<0.01) and dose-dependently restored these ions levels: 140.00 ± 4.08, 5.11 ± 0.07, 3.12 ± 0.09, and 107.98 ± 1.36 mmol/L, respectively, for Na^+^, K^+^, Ca^++^, and Cl^−^ (Table 3).

### 3.9. Effect of* Crinum jagus* Water/Ethanol Extract on* Shigella flexneri* Diarrheic Rats' Blood Cell Count

Blood cell count in diarrhea control rats (DC) showed a reduction of WBC (2.74 ± 0.75 X 10^3^/mm^3^) and haemoglobin (11.16 ± 0.80 g/dL) and an increase of RBC (6.42 ± 0.45 X 10^6^ / mm^3^) and platelets (374.80 ± 2.05 X 10^6^ / mm^3^) compared to normal rats. Hematocrits (Ht), mean globular volume (MGV), mean globular haemoglobin (MGH), and mean corpuscular haemoglobin concentration (MCHC) were, respectively, 40.04 ± 1.76%, 59.98 ± 2.06 *μ*m^3^, 34.02 ± 3.79, and 31.56 ± 2.07 *ρ*g/dL in normal control (Table 4). Water/ethanol* C*.* jagus* extract significantly (p<0.05) and dose-dependently increased Ht (44.40 ± 0.86%) and MCHC (35.04 ± 0.97 *ρ*g/dL) compared to diarrheic control (Table 4).

### 3.10. Intestine Morphology of* Shigella flexneri*-Infected Rats after Treatment with* C. jagus* Water/Ethanol Extract and Ciprofloxacin

**Figure 6:**
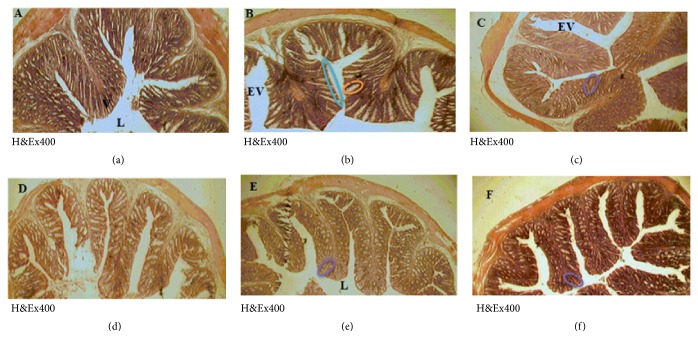
Histology of the colon in normal rats (a), diarrheic control rats (b), and rats treated with* C. jagus* water/ethanol extract of 25 mg/kg (c), 50 mg/kg (d), and 100 mg/kg (f) and with ciprofloxacin (2.5 mg/kg) (e). Light blue color: abrasion of the colon epithelium; orange color: mucus-filled goblet cell; purple color: goblet cell free from mucus; (L) colon lumen.

Control rat colon presented epithelium with normal and large folds and microfolds (V) (Figure 6(a)). Diarrheic control animal colon (Figure 6(b)) showed abrasion and erosion of the mucosal lining, giving rise to large interfolds space (EV) and a great number of mucus-filled goblet cells.* C*.* jagus* water/ethanol extract of 25 mg/kg (Figure 6(c)), 50 mg/kg (Figure 6(d)), and 100 mg/kg (Figure 6(f)) and ciprofloxacin (2.5 mg/kg) (Figure 6(e)) dose-dependently restored the epithelium structures, with well-reconstituted folds and microfolds, and reduced the density and the size of mucus-filled goblet cell.

### 3.11. Acute Toxicity

One hour after extract administration (5000 mg/kg), rat became less motile and exhibited some abdominal contractions. These signs lasted for about 30 min, and, within the 14 observation days, no behavioral trouble and signs of toxicity were noticed. Normal stools were emitted, and no death was recorded. Median lethal dose of this extract may be greater than 5 000 mg/kg BW, and the extract may be weakly toxic.

## 4. Discussion

*In vitro*,* Crinum jagus* extract showed concentration-dependent antibacterial activities. The MIC/MBC ratio was lower than 4 and could thus indicate a bactericidal activity [26]. This activity was further confirmed by the reduced bacterial load observed with* in vivo* studies. In diarrheic control rats,* Shigella flexneri* induced diarrhea characterized by soft, liquid, mucus linked, and bloody stools, emitting a fetid odor. Bloody stool reflects the invasion or destruction of the epithelial cells of the host's intestine, resulting in ulceration and hemorrhage. Furthermore, an increase in bacterial load, weight loss, interleukin-2, interferon gamma, immunoglobulins A and M, and motilin blood levels and a decrease in VIP, K^+^, Na^+^, and Cl^−^ ions blood concentrations and white blood and red blood cells were observed. As in diarrheal patients, diarrheal rats showed increased motilin blood levels. Motilin induces intestine smooth muscle contraction via its receptors located on circular smooth muscle [27, 28] or indirectly by activation of serotonin 5-HT_3_ receptors [29]. Bacterial antigens, particularly lipopolysaccharides (LPS), are responsible for the inflammation of colon mucosa with, as consequence, the abundant fluid accumulation in the small intestine [30, 31]. This fluid content, particularly, in acid or alkali and in fat which are potent secretagogue for motilin may have increased plasma motilin level [32]. Treatment with* Crinum jagus* ethanol/water extract may have decreased bacterial load, hence LPS concentration, and therefore fluid accumulation, which resulted in a decrease of plasma motilin concentration.

VIP (vasoactive intestinal peptide) is a neurotransmitter that inhibits the contraction of intestine smooth muscles. Its action here is closely linked to that of nitric oxide (NO), another intestine inhibitory neurotransmitter, so that the release of one leads to the release of the other [33].* Crinum jagus* may have stimulated the production or secretion of VIP, since its administration to rats induced an increase of this hormone. The increase in red and white blood cells observed in extract treated animals may indicate that* Crinum jagus* like many other plants has immunomodulatory properties [34].

Morphologically, the colon of diarrheal rats showed an eroded epithelium, folds with large interfolds spaces, and abrasions, giving them a flat appearance with many goblet cells filled with mucus. Mucus production is a defense reaction of the digestive tract and it occurs whenever the mucosa integrity is endangered.* Crinum jagus* ethanol/water extract and ciprofloxacin protected colon morphology and restored the epithelium to its normal appearance with large folds and microfolds with many well-reconstituted digits and goblet cells where mucus density has decreased considerably.

Increase in* Shigella* load and the quantity of glary bloody diarrheal stools observed in diarrheic control rats may be a consequence of bacterial overgrowth and, hence, Shiga toxin production, destruction of defense cells, and intestinal tissue. All these are typical signs of infectious diarrhea with invasive germs [35]. Shiga toxin is responsible for intestinal mucosa inflammation, the consequences of which include increase in motilin production and alteration of electrolytes absorption. This may thus explain the decrease observed in K^+^, Ca^++^, Na^+^, and Cl^−^ concentrations in nontreated diarrheal animals [35]. In these control animals, increase of interleukin-2, interferon gamma, and immunoglobulins A and M may be seen as a defense reaction against* Shigella* overgrowth. Diarrheal animals treated with extract or ciprofloxacin showed normal stools with no blood or mucus and a decrease in* Shigella* load. This drop in* Shigella* load could have resulted from direct action of some* Crinum jagus* bioactive compounds such as phenolic compounds on the bacteria. Phenolic compounds in fact were shown to induce rupture of bacteria plasma membrane and increase its permeability [36]. Decrease observed in* Shigella* load can be thought not to be a result of the immune system activity, since treatment with extract or ciprofloxacin did not induce significant increase of interleukin-2, interferon gamma, and immunoglobulins A and M blood levels more than what was observed in untreated diarrheal animals.

Colonization of the digestive mucosa by bacteria, including* Shigella* and* Salmonella*, is followed by activation of the lymphoid tissue and, hence, payer's patches and gut associated lymphoid tissue (GALTs), as well as significant immunoglobulins and cytokines production [36, 37]. IgA, the most important immunoglobulin produced in the intestine and other mucosal surfaces, is the first defense line against bacteria and viruses [38]. It inhibits microbe adherence and mucosal surfaces colonization, neutralizes microbial toxins, limits microbial growth, and activates the expulsion of microbial plasmids [39]. IgM activates the complement system and contributes to the agglutination and neutralization of bacteria and viruses and is also necessary in the induction of memory B cells [40]. IL-2 activates T and B cells maturation and immunoglobulin production [41]. INF-gamma by activating macrophages and T helper cells causes phagocytosis and intracellular bacteria destruction. It also activates the expression of the adhesion factor VCAM-1 of the intestinal epithelium, which binds microbes for destruction [37]. The extract or ciprofloxacin relatively decreased immunoglobulin and cytokine levels in diarrheal animals. This may be due to a decrease in the stimulation of lymphoid systems or immunoglobulins production, following bacteria destruction or elimination.

*Crinum jagus* extracts protected diarrheal animals against weight loss. The weight gain caused by the plant extract may result from the stimulation of the appetite or the reduction of water and electrolyte losses.

## 5. Conclusion

*In vivo*,* Crinum jagus *extract inhibited bacterial growth and was found to be bactericidal. It prevented animal from glary bloody stools and decreased bacterial load in infected treated rats. Furthermore,* C*.* jagus *increased vasoactive intestinal peptide, decreased motilin productions, and restored colonic epithelium. These results support the use of the plant by traditional healers in the treatment of infectious diarrhea and could therefore be used for production, in collaboration with chemists and pharmacists, of improved forms of traditional medicines that will be used by population for effective treatment of shigellosis. In future studies, detailed phytochemical profiles of the extract would be determined to elucidate the major compounds underlying the reported antidiarrheic effect.

## Figures and Tables

**Table 1:** *Shigella flexneri*'s sensitivity to *Crinum jagus* ethanol/water extract and ciprofloxacin.

Treatment	Concentrations (mg/mL)	Inhibition diameter (mm)
*Crinum jagus* extract	200.00	25.36 ± 0.65
	100.00	24.53 ± 0.07
	50.00	23.21 ± 0.08
	25.00	22.47 ± 0.01
	12.50	21.91 ± 0.26
	6.25	21.59 ± 0.30
	3.13	21.40 ± 0.33
	1.56	20.50 ± 0.40
	0.78	19.78 ± 0.18
	0.39	18.90 ± 0.12

Ciprofloxacin	0.03	28.00 ± 0.30

**Table 2:** Minimum inhibitory concentration (MIC) and minimum bactericidal concentration (MBC) of *Crinum jagus* water/ethanol extract and ciprofloxacin on *Shigella flexneri*.

Inhibition parameters	*Crinum jagus*	Ciprofloxacin
Minimum inhibitory concentration (mg/mL)	0.10	2.0X10^−3^
Minimum bactericidal concentration (mg/mL)	0.30	4.0X10^−3^
MBC/MIC ratio	3.0	2.0

**Table 3:** Sodium, potassium, calcium, and chloride plasma levels in rats infected with *Shigella flexneri* after treatment with *C*. *jagus* water/ethanol extract and ciprofloxacin.

Ions	NC	DC	Cipro	WECj25	WECj50	WECj100
Na^+^	143.33 ± 4.08	93.33 ± 4.08*∗∗*	146.67 ± 6.24^**a**^	120.00 ± 6.24**∗**^**a**^	136.67 ± 6.24^**a**^	140.00 ± 4.08^**a**^
K^+^ (mmol/L)	5.11 ± 0.09	3.75 ± 0.08*∗∗*	5.46 ± 0.12**∗∗**^**a**^	4.32 ± 0.09**∗∗**^**a**^	5.00 ± 0.08**∗∗**^**a**^	5.11 ± 0.07**∗∗**^**a**^
Ca^++^ (mmol/L)	3.25 ± 0.01	2.44 ± 0.03*∗∗*	3.06 ± 0.04^**a**^	2.52 ± 0.01**∗∗**	2.80 ± 0.11*∗∗*	3.12 ± 0.09^**a**^
Cl^−^ (mmol/L)	113.83 ± 2.29	95.74 ± 1.46*∗∗*	108.51 ± 2.29^**a**^	100.00 ± 1.36**∗∗**	104.79 ± 1.36**∗∗**^**a**^	107.98 ± 1.36^**a**^

WECj25, WECj50, and WECj100: *C*. *jagus* water/ethanol extract of 25, 50, and 100 mg/kg; Cipro: ciprofloxacin 2.5 mg/kg; NC: normal control; DC: diarrheic control; Na^+^: sodium ions; K^+^: potassium ions; Ca^++^: calcium ions; Cl^−^: chloride ions (n=5). Significant difference: *∗∗*p<0.01 compared to normal control (NC); ^a^p<0.01 compared to diarrheic control (DC).

**Table 4:** Blood cell count in rats infected with *Shigella flexneri* after treatment with *C*. *jagus* water/ethanol extract and ciprofloxacin.

Groups	NC	TD	Cipro	WECj25	WECj50	WECj100
WBCX10^3^/mm^3^	5.24 ± 0.14	2.74 ± 0.75*∗∗*	3.92 ± 0.22	4.79 ± 0.08^b^	4.92 ± 0.55^b^	5.22 ± 0.32^a^
RBCX10^6^/mm^3^	4.28 ± 0.19	6.42 ± 0.45	7.06 ± 0.35*∗*	7.82 ± 1.19*∗∗*	7.37 ± 0.51*∗∗*	8.25 ± 0.39*∗∗*
Hb (g/dL)	13.15 ± 0.76	11.16 ± 0.80	12.18 ± 0.93	12.32 ± 0.74	13.14 ± 0.54	12.30 ± 0.62
Ht (%)	40.04 ± 1.76	34.32 ± 1.93	36.68 ± 2.25	40.52 ± 1.67	42.64 ± 3.30^b^	44.40 ± 0.86^b^
PcX10^6^/mm^3^	373.40 ± 1.13	374.80 ± 2.05	361.80 ± 4.72^b^	485.64 ± 3.02*∗∗*^a^	497.20 ± 2.15*∗∗*^a^	826.60 ± 2.89*∗∗*^a^
MGV (*μ*m^3^)	59.98 ± 2.06	52.20 ± 0.53*∗*	51.80 ± 0.73*∗∗*	54.00 ± 0.84*∗*	52.20 ± 0.86*∗*	53.20 ± 0.97*∗*
MGH **(***ρ*g)	34.02 ± 3.79	17.40 ± 0.08*∗∗*	17.40 ± 0.22*∗∗*	14.26 ± 2.81*∗∗*	16.60 ± 0.97*∗∗*	14.56 ± 1.39*∗∗*
MCHC (*ρ*g/dL)	31.56 ± 2.07	33.46 ± 0.38	33.46 ± 0.47	33.78 ± 0.84	34.74 ± 0.66	35.04 ± 0.97

WECj25, WECj50, and WECj100: *C*. *jagus* water/ethanol extract of 25, 50, and 100 mg/kg; Cipro: ciprofloxacin 2.5 mg/kg; NC: normal control; DC: diarrheic control; WBC: white blood cell; Hb: haemoglobin; Ht: hematocrit; RBC: red blood cell; Pc: platelets; MGV: mean globular volume; MGH: mean globular haemoglobin; MCHC: mean corpuscular haemoglobin concentration (n=5). Significant difference: *∗*p<0.05 and *∗∗*p<0.01 compared to normal control (NC); ^a^p<0.01 and ^b^p<0.05 compared to diarrheic control (DC).

## Data Availability

The data used to support the findings of this study are available from the corresponding author upon request.
